# Negative Air Ions and Their Effects on Human Health and Air Quality Improvement

**DOI:** 10.3390/ijms19102966

**Published:** 2018-09-28

**Authors:** Shu-Ye Jiang, Ali Ma, Srinivasan Ramachandran

**Affiliations:** Temasek Life Sciences Laboratory, 1 Research Link, National University of Singapore, Singapore 117604, Singapore; shuye@tll.org.sg (S.-Y.J.); zhigang@tll.org.sg (A.M.)

**Keywords:** negative air ions, superoxide, particulate matter, pulsed electric field

## Abstract

Negative air ions (NAIs) have been discovered for more than 100 years and are widely used for air cleaning. Here, we have carried out a comprehensive reviewing on the effects of NAIs on humans/animals, and microorganisms, and plant development. The presence of NAIs is credited for increasing psychological health, productivity, and overall well-being but without consistent or reliable evidence in therapeutic effects and with controversy in anti-microorganisms. Reports also showed that NAIs could help people in relieving symptoms of allergies to dust, mold spores, and other allergens. Particulate matter (PM) is a major air pollutant that affects human health. Experimental data showed that NAIs could be used to high-efficiently remove PM. Finally, we have reviewed the plant-based NAI release system under the pulsed electric field (PEF) stimulation. This is a new NAI generation system which releases a huge amount of NAIs under the PEF treatment. The system may be used to freshen indoor air and reduce PM concentration in addition to enriching oxygen content and indoor decoration at home, school, hospital, airport, and other indoor areas.

## 1. Introduction

Negative air ions (NAIs) have been discovered for more than 100 years [1]. Now, NAI generators are widely available for home or industrial uses. In the meantime, various new technologies were developed and used to further improve NAI generation and reduce the release of its byproduct ozone. However, some controversial results or comments have been reported for the beneficial effect on humans/animals or on reductions in bacterial densities. Here, we have carried out a comprehensive reviewing on NAIs. On the other hand, strong evidence had shown the roles of NAIs in high-efficiently reducing particulate matter (PM) concentration. Thus, more work should be done to further improve NAI release by new methods or devices so that NAIs could be more widely used for air cleaning. Here, we review the generation of NAIs and their effects on humans, animals, and microorganisms. We then discussed the involvement of superoxide ions in biological effects of NAIs. Subsequently, we focused on plant-based NAI generation systems as these are relatively new NAI generation systems with some advantages on traditional corona discharge NAI generators. We also reviewed the air cleaning ability of NAIs, especially in removing PM with diameters less than 10 micrometers (PM_10_).

## 2. Systematic Review of Literatures

The systematic review of studies on NAIs initiated by literature searches. Three databases were selected including PubMed database (available online: https://www.ncbi.nlm.nih.gov/pubmed/advanced), ScienceDirect database (available online: https://www.sciencedirect.com/search/advanced), and IEEE Xplore Digital Library (available online: https://ieeexplore.ieee.org/search/advsearch.jsp). The coverage period of the database search is around 100 years from 1918 to 2018 (the updated search date is 10 September 2018). Three keywords “negative”, “air”, and “ion” were used to search all collected articles in these databases. The database searches harvested a total of 335, 681, and 221 articles that contain all of the three keywords in either titles or abstracts when the PubMed database, ScienceDirect, and IEEE Xplore databases were employed, respectively. Only peer-reviewed and English-language articles were considered. We then screened these articles by manually reviewing titles, which led to 170, 279, and 117 studies selected (duplicated references were excluded); the remaining were excluded due to no relationship to the NAI topic. We further carried out abstract screening to exclude more unrelated articles. Based on the abstract reviewing, 93, 113, and 57 references were selected, which were subjected to full-length reviewing. During full-length article reviewing, some cited references, which were not included in the full-length review list, were also selected for additional reviewing.

## 3. Negative Air Ions and Their Generation

Air ions are electrically charged molecules or atoms in the atmosphere [2]. An air ion is formed when a gaseous molecule or atom receives sufficiently high energy to eject an electron [3]. NAIs are those that gain an electron, while positive air ions lose an electron. The natural and artificial energy sources include (1) radiant or cosmic rays in the atmosphere; (2) sunlight including ultraviolet; (3) natural and artificial corona discharge including thunder and lightning; (4) the shearing forces of water (Lenard effect); (5) plant-based sources of energy.

### 3.1. Radiant or Cosmic Rays in the Atmosphere

The radioactive elements such as uranium, radium, actinium, and thorium widely exist in our planet. They decay in the atmosphere and emit α, β, and/or γ rays, which ionize the air. Thus, radiant and cosmic ray ionization is ubiquitous in the Earth’s atmosphere. The cosmic ray ionization accounts for around 20% of the total ionization over land surfaces [4]. They are also the principal energy sources that generate NAIs over the oceans [5]. The concentration of NAIs produced by these rays might reach from 500 ions per cm^3^ in land surface [6] to more than 1000 ions per cm^3^ at 15 km away from the land surface [7].

### 3.2. Sunlight Including Ultraviolet

The photoelectric effect is the emission of electrons when a certain wavelength of light is shone onto a metallic surface. NAIs are generated by accepting these emitted electrons. The photoelectric effect may contribute less to the NAI generation as only some wavelength of lights shows the ability to emit electrons by lighting. One of the examples is the negative ion generator using an ultraviolet source to irradiate electrically conductive material, which was patented as early as 1964 (Patent No. US 3128378 A). In this patent, an ultraviolet lamp was used to irradiate metal materials, which photo-electrically eject electrons. The electrons then collide with air molecules and generate NAIs.

**Figure 1:**
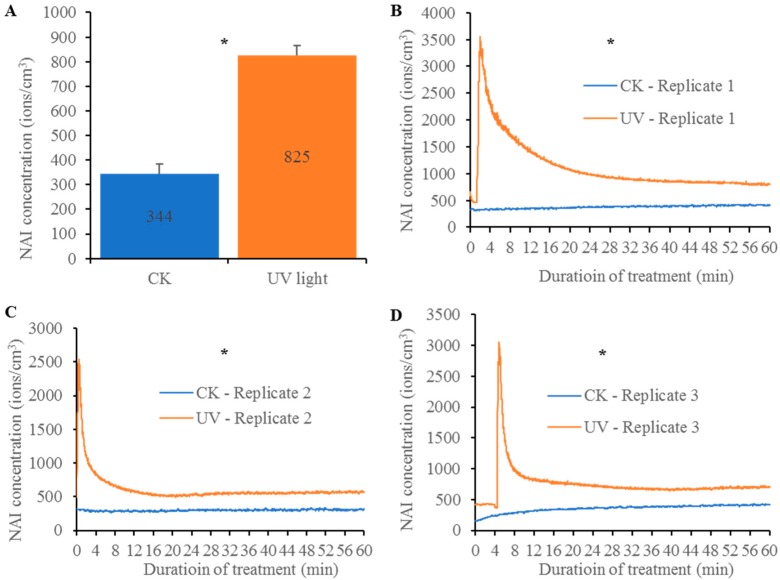
NAI generation by UV lighting. The experiment was carried out in a growth chamber with dimensions of 80 cm length, 80 cm width and 80 cm height. A 30 watts of UV light (UV-C, 100–280 nm) was provided by Safer Electric Ltd., Singapore. NAI concentration was measured by the DLY-4G-232 air ion counter (Kilter Electronic Institute Co., Ltd., Zhangzhou, Fujian Province, China). (**A**) The average NAI concentrations of control (CK, no UV light) and UV lighting. The NAI concentration under UV light was significantly higher than the control as indicated by Mann–Whitney U test at *p* < 0.00001. (**B**–**D**) Graphs to show the curves of NAI concentrations among three different replicates. The star “*” indicated that the NAI concentration under UV lighting was statistically higher than that in CK by Mann–Whitney U test at *p* < 0.00001. NAI: negative air ions.

On the other hand, NAIs can be generated by a certain wavelength of lights through directly ionizing air molecules. For example, ultraviolet (UV) can be used to directly ionize air molecules to generate NAIs [5,8]. Actually, UV-mediated ionization is the dominant NAI sources in the above 60 km altitude of atmosphere [5]. These highly concentrated NAIs from UV in the upper layers of the atmosphere are diffused to the ground surface at low speeds. Ionization by UV radiation is not a major contributor of NAIs in the lower atmosphere due to the low dose of UV rays available in this layer [5]. Although reports showed that the UV rays significantly mediated the air ionization, little systematical study was carried out on the effect of artificial UV light on NAI generation. We carried out an experiment to investigate the contribution of UV light to the generation of NAIs (Figure 1; Table S1). The experiment was carried out in a growth chamber with 80 cm length × 80 cm width × 80 cm height and detailed description was provided in the Supplementary Experiment. NAIs were measured under UV light conditions with the normal light condition as a control. The data from three replicates of experiments showed that UV lights indeed promoted the NAI generation. In the chamber, the average NAI concentration was 344 ions/cm^3^ within one hour. The NAI concentration was increased to 825 ions/cm^3^ under UV light condition, significantly higher than the control (Figure 1A). Further observation showed that there were peaks of NAI generation within 8 min after UV lighting (Figure 1B–D; Table S1). After the peaks, NAI concentration was kept at a relatively stable value but was still higher than the control. For all three replicates or each replicate, a nonparametric two-tailed Mann–Whitney U test was carried out as described in the Supplementary Experiment and the statistical analysis showed that NAI concentrations under UV lighting conditions in all three replicates were significantly higher than those under normal lighting conditions (CK) with *p* < 0.00001. The analysis further confirmed the promoting effect of UV lighting on NAI generation. Thus, our experiment showed that UV lighting could be used to generate NAIs. However, only low concentrations of NAIs were generated under our UV light conditions.

### 3.3. Natural and Artificial Corona Discharge Including Thunderstorms and Lightning

The atmosphere surrounding the earth is subjected to a natural electric field and its intensity is continuously fluctuating under both local and global influences [9]. The local influences include geographical location and weather conditions such as thunderstorms, rain, fog, mist, and so on; the global facts refer to classical daily electric field variations [4]. When leaf points or branches of trees have a high potential difference from their surroundings in their electric fields, corona discharge (also called point discharge) occurs and NAIs may be released [5,10]. Generally, corona discharge occurs at the atmosphere conditions under high average electric fields [5]. For example, in a mountain area, high electric fields and low atmospheric pressure promote the onset of corona discharge [11]. Thunderstorms and lightning will generate very high electric field conditions and corona discharge subsequently occurs. Therefore, NAIs will be released at a huge amount after thunderstorms and lightning. However, released NAIs will be gradually decayed with the discontinuous thunderstorms. In addition to thunderstorms and lightning, mist may also contribute to NAI generation. In a forest, electric field variations were observed during the mist formation and dissipation, which may trigger corona discharge and NAI generation [9].

Artificial corona discharge is an efficient way to generate NAIs. When a high negative voltage is applied to a conductor/electrode and generated electric field is high enough, corona discharge occurred [12,13]. If a charged conductor/electrode has a needle-type with a sharp point, the electric field around the tip will be significantly higher than other parts and air near the electrode can become ionized and NAIs are generated [14]. Intensity of corona discharge depends on the shape and size of the conductors as well as applied voltage. Irregular conductor, especially with a sharp point, gives rise to more corona than a smooth conductor and large-diameter conductors produce lower corona than small-diameter conductors; the higher the voltage applied, more NAIs are generated [14,15]. The closer the distance to corona point, the higher NAI concentration is detected as continuous generation of NAIs by corona discharge is related to a chain reaction process called an electron avalanche [16]. The application of artificial electric field and corona discharge on plants was carried out as early as the 1960s [17,18]. Bachman and Hademenos (1971) showed that under high voltage, artificially applied electrical fields near the pointed barley leaf tips were intensified [19] and as a result, corona discharge occurred and air ions and ozone were generated. Studies mainly focused on biological effects such as growth response, evaporation, and plant damage as well as the effects of generated ozone and NAIs on plant growth [11,18,19,20,21,22].

### 3.4. The Shearing Forces of Water (Lenard Effect)

The considerable numbers of NAIs are found under waterfalls or in the seashores. These NAIs are generated by Lenard effect. Lenard effect was also called spray electrification or waterfall effect and was first systematically studied by Philipp Lenard [23], who won the Nobel Prize for Physics in 1905 for his research on cathode rays and the discovery of many of their properties. The study showed that NAIs were generated from the surrounding air molecules by charging themselves negatively when water droplets collide with each other or with a wetted solid to form fine spray of drops. The study also showed that several factors may affect the degree of charge separation in spray processes and, therefore, may affect the generation and concentration of NAIs. These factors include water drop temperature, dissolved impurities, speed of the impinging air blast, and foreign impinging surfaces of droplets. Based on the “Lenard effect”, water shearing appliance has been designed to generate NAIs [24]. Water shearing produced only superoxide ions (O_2_^−^) which was bound to clusters of water molecules to form the structure O_2_^−^(H_2_O)_n_ [25], and was essentially regarded as a natural source of NAIs [24]. NAIs generated by the “Lenard effect” might improve erythrocyte deformability, thereby aerobic metabolism [24].

### 3.5. Plant-Based NAI Release under Normal Growth Conditions and by Pulse Electric Stimulation

Plants were reported to have the ability to generate NAIs under normal growth conditions and have been regarded as natural resources for NAI generation [26,27]. Different plants released different amounts of NAIs under natural growth conditions (Table 1). However, under normal growth conditions, plants released very low concentration of NAIs (<200 ions/cm^3^, Table 1). Bachman and Hademenos (1971) reported the NAI generation by applying high-voltage electric field to plants [19]. Later, Tikhonov et al. (2004) showed that plants could release huge amounts of NAIs under pulse electric field (PEF) stimulation [28]. Since then, several other studies were carried out to investigate the effect of PEF stimulation on plant NAI generation (Table 2). Generally, under natural growth conditions, plants release less than 200 ions/cm^3^ (Table 1). However, after PEF stimulation, more than 3.5 × 10^6^ ions/cm^3^ were detected (Table 2). Several parameters may affect the NAI release under PEF stimulation including plant species and output voltages in PEF stimulation (Table 2) as well as light intensity, temperature, pulse interval, and the pulse width of PEF [28,29,30]. Studies of plant morphology on NAI release showed that species with blade shapes generated higher concentration of NAIs [27]. All these studies may provide an alternative to artificially generate NAIs through a plant-based method.

Besides the abovementioned 5 different sources of NAIs, some other ways also may generate NAIs. For example, the friction by rapidly moving great volumes of air over land may generate NAIs and storms have been also regarded as an energy source for NAI generation [31].

### 3.6. The Fate of Generated NAIs

As described above, we have reviewed the generation of various sources of NAIs and various factors affecting NAI generation. Here, we further explored the fate of generated NAIs. Generally, generated NAIs are not stable and will be gradually decayed. When NAIs combined with water molecules and form negative-ion clusters, their lifetime will be longer. For example, the half-life of negative oxygen ions O_2_^−^(H_2_O)_n_ by the Lenard effect is around 60 seconds, but the NAIs generated by corona discharge could survive only several seconds [32]. Such a trend in survival duration of these two different sources of NAIs was also observed by another study [33]. In the study, the lifetime of NAIs generated by the Lenard effect was several minutes, significantly longer than that of electrically generated NAIs. However, other studies showed that the lifetime of NAIs in clean air is around 100 s ([34] and references therein). In fact, several other factors including the concentrations of NAIs and aerosols, and the intensity of electric fields may affect lifetimes of NAIs [35]. This may explain why different lifetimes were reported by several different references as mentioned above.

## 4. Plants as a Source for Generating NAIs

Negative oxygen ions are the most commonly recognized NAIs. Reports showed that superoxide O_2_^•−^ was a kind of NAIs [28,36,37,38]. Among NAIs generated by natural atmosphere and the Lenard effect (waterfall), superoxide O_2_^•−^ are the major negative ions [37,38]. In plants, superoxide O_2_^•−^ is mainly produced in the thylakoid membrane of photosystem I (PSI) [39]. Generation of superoxide O_2_^•−^ within PSII has also been reported [40,41,42]. Some plant peroxidases are located in the apoplast by ionically or covalently bonding to cell wall polymers and these cell wall peroxidases might contribute to the generation of extracellular superoxide ions [43,44]. Different plant species might exhibit the difference in superoxide generation. For example, high rates of extracellular superoxide were generated in bryophytes and lichens [45]. In addition, generation of superoxide could be regulated by some external stimulation. For example, in chloroplasts, through treating plants with methyl viologen (MV) or paraquat under light conditions, it is possible to generate O_2_^•−^ steadily by univalent reduction of MV or paraquat and subsequently transferring their electrons to oxygen [39,46,47]. Immediately induced and transient generation of extracellular superoxide anion was observed after adding salicylic acid (SA) to tobacco (*Nicotiana tabacum*) suspension culture [48].

Furthermore, the generation of superoxide in plant leaves can be improved by regulating gene expression. For instance, expression of the gene encoding Rho-related small G protein (ROP) 2 promoted superoxide generation in *Arabidopsis* leaf extracts [49]. Interestingly, the production of superoxide in plants could be improved by transgenic techniques. Rice *PsbS1* gene encodes a 22-kDa Photosystem (PS) II protein involving in nonphotochemical quenching (NPQ) of chlorophyll fluorescence. Disfunction of this gene by either transfer DNA (T-DNA) knockout or RNAi led to increased superoxide production [50]. The result provides a new way to improve plants in their superoxide release.

In addition to superoxide, other negative ions also contribute to the composition of plant NAIs. However, little is known on how these negative ions are generated and released in plants. Studies mainly focused on several factors that affect the release of NAIs in plants. Wang and Li (2009) investigated the effects of light intensities on NAI release in five plant species (*Aloe arborescens*, *Clivia miniata*, *Chlorophytum comosum*, *Opuntia brunnescens*, *Crassula portulacea*) [27]. Their data showed that the NAI release was significantly increased with the enhancement of light intensities for *Aloe arborescens*. However, for the remaining 4 plant species, less effect was observed and NAI release was not significantly affected by light intensities. Skromulis and Noviks (2012) surveyed the effects of meteorological factors on NAI release in a city and found that the highest level of NAI concentration was observed in morning sections and less air pollutants were detected in the duration [51]. Wu et al. (2017) analysed the NAI release of three plant species, *Crinum asiaticum*, *Narcissus tazetta*, and *Zephyranthes carinata*, under different light intensities and found that the stronger light might stimulate the plants to release more NAIs [30].

## 5. Composition of Negative Air Ions

**Figure 2:**
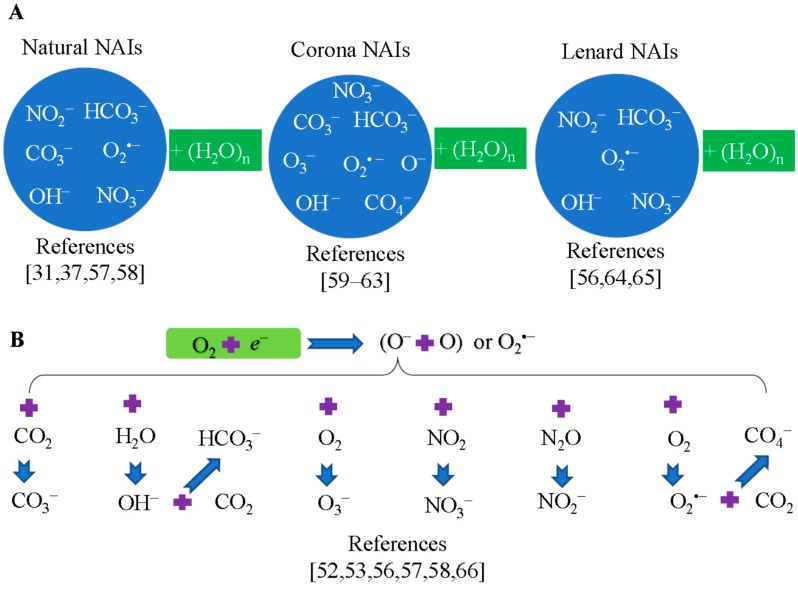
NAI species generated by different methods and evolution of oxygen-based NAIs. There were some differences in NAI compositions from different studies as their experimental conditions might vary. (**A**) NAI compositions. (**B**) The evolution of oxygen-based NAIs. The blue arrows indicate the NAI transformation processes. Please refer to the cited paper for more details [31,37,52,53,56,57,58,59,60,61,62,63,64,65,66].

Generally, NAIs are composed of multiple negatively charged molecules and these negative ions combine with several or up to 20 or 30 water molecules and form negative-ion clusters such as CO_3_^−^(H_2_O)_n_, O^−^(H_2_O)_n_, and O_3_^−^(H_2_O)_n_. [24,52,53]. Mass spectrometric techniques were widely used to determine the composition of NAIs from various sources [54,55,56]. Early measurements suggested that the majority of lower tropospheric negative ions would be composed of O_2_^−^, CO_3_^−^, or NO_3_^−^ and their (H_2_O)_n_ clusters as well as HSO_4_^−^ core ions [5,57]. Studies showed that atmosphere NAIs also included additional ions such as OH^−^, NO_2_^−^, HCO_3_^−^ and their water clusters [31,57]. Figure 2 summarizes the composition from various sources of NAIs and the evolution of oxygen-based NAIs [31,37,52,53,56,57,58,59,60,61,62,63,64,65,66].

Negative ion species generated by corona discharge have been identified through mass spectrometry by several experiments [55,66,67]. The majority of negative ions are CO_3_^−^ and other negative ions include O^−^, O_3_^−^, NO_3_^−^, and so on, which consist of less than 10% [52,67,68,69,70,71,72,73,74]. Reports from Nagato et al. (2006) showed that negative ion compositions are different at different reaction times of corona ionization as observed by mass spectrometry [74]. Based on their results, NO_3_^−^ is the major ion followed by HCO_3_^−^ and others. Both results from Shahin (1969) and Nagato et al. (2006) indicated that O_2_^−^ is not dominant products of negative ions produced by corona discharge [67,74]. The majority of NAIs from corona discharge were listed in Figure 2A.

Waterfalls-induced negative ions evolved from both O_2_^−^ and O^−^ [56]. The OH^−^ concentration was significantly increased near waterfalls. These three ions, O_2_^−^, O^−^, and OH^−^, further evolved into other types of ions. As a result, the following 5 types of ions were regarded as the major negative air ions generated by waterfalls. They were listed in Figure 2A. For the plant-based NAIs released by PEF stimulation, no reports were available on their ion composition.

Based on the studies mentioned above, NAIs may evolve from one NAI to another NAI. For example, the NAI O^−^ is formed when an oxygen molecule O_2_ obtains an electron (Figure 2B). The NAI O^−^ may contribute to the formation of secondary NAIs by collision-aided electron attachment processes when other molecules exist in the same space [58]. As a result, other NAIs are generated such as O_2_^−^, CO_4_^−^, CO_3_^−^, OH^−^, HCO_3_^−^, O_3_^−^, NO_3_^−^, and NO_2_^−^ (Figure 2B). Parts et al. (2007) described a more complicated evolutionary process of NAIs [56]. In fact, the NAI evolution is related to the surrounding air composition. NAIs are continually changing as they collide with molecules in the air. Thus, NAIs are dynamic in their composition, which depends on ionization potential and electron affinity, the proton affinity, the dipole moment and the polarizability as well as the reactivity of the molecule [75].

## 6. Biological Effects of Negative Air Ions on Human/Animal Health and Microorganism Growth as well as Plant Development

Krueger and Reed (1976) reviewed key reports published in that period on the biological effects of NAIs [1]. They proposed the serotonin hypothesis of NAI biological actions. Serotonin is a very powerful and versatile neurohormone. It functions in inducing profound neurovascular, endocrinal, and metabolic effects in humans or animals. It plays important roles in basic patterns of life including sleep and mood regulation. Evidence has shown that NAIs could significantly reduce the level of serotonin in blood or brain, etc. [1,76,77]. Subsequent evidence showed that superoxide ions have been involved in the biological effects of NAIs [37] and in vitro experiments demonstrated that serotonin could be oxidized into tryptamine-4,5-dione by superoxide ions [78]. Thus, some of the biological effects of NAIs were related to the reduction of serotonin. However, others reported no significant effect of NAIs on the concentration or turnover of serotonin in rats under their exposure conditions [35,79]. Bailey et al. (2018) carried out a comprehensive review on the effects of air ions on serotonin and other neurotransmitters and their analysis indicated some modest or strong evidence to support no effect hypothesis [35].

There are many references to report the possible biological effects [24,32,33,35,38,76,77,78,79,80,81,82,83,84,85,86,87,88,89,90,91,92,93,94,95,96,97,98,99,100,101,102,103,104,105,106,107,108,109,110,111,112,113,114,115,116,117,118,119,120,121,122,123,124]. And some of them were listed in Table S2. The negative oxygen ion concentration exceeding 1000 ions/cm^3^ has been regarded as the threshed value for fresh air and the concentration should be higher for boosting the human immune system ([80] and references therein). Exposure to NAIs exhibit wide effects on animal/human health, anti-microorganisms and plant growth (Table S2). The effects of NAIs on human/animal health mainly focused on the cardiovascular and respiratory system as well as on mental health (Table S2). The effects of NAIs on the cardiovascular system included improving erythrocyte deformability and aerobic metabolism [24] as well as decreasing blood pressure [32,77,81,82]. However, no effect was also reported on blood pressure [83] or heart rate [84,85] and related data on cardiovascular function studies were not quantitatively evaluated [35]. On mental health, exposure to NAIs showed highly significant increase in performance of all tested tasks (mirror drawing, rotary pursuit, visual reaction time, and auditory) [86], alleviating symptoms of seasonal affective disorder (SAD) [87]. Similar effects of NAIs on relieving symptoms in mood disorders to antidepressant nonpharmacotherapy trials were observed [38]. NAIs also showed effective treatment of chronic depression [88]. On the contrary, no effect of NAIs on mental health was reported in other studies [89,90]. Comprehensive analysis on literatures showed no conclusive results on the potential therapeutic effects of NAIs on depression [35]. As for the effect of NAIs on respiratory function, exposure to negative or positive air ions does not appear to play an appreciable role in respiratory function [91]. In addition, some reports also showed the effects of NAIs on inhibiting cancer cells. For example, water-generated NAIs activated natural killer (NK) cell and inhibit carcinogenesis in mice [33]. The presence of NAIs is credited for increasing psychological health, productivity, and overall well-being [38,92,93]. Reports also showed that NAIs attached themselves to particles such as dust, mold spores, and other allergens [37]. As a result, people in the NAI atmosphere relieved symptoms of allergies to these particles. Generally, although some reports showed that air enriched with NAIs have multiple beneficial therapeutic effects in normalizing arterial pressure and blood rheology, supporting tissue oxygenation, easing stress conditions, and augmenting resistance to adverse factors [94], a systematic review suggested no consistent or reliable effects of NAIs on cardiovascular and respiratory system as well as on mental health [35].

Similarly, there are lots of research articles to present the effects of NAIs on the growth of microorganism (Table S2). The majority of the studies focused on bacteria and the presence of high concentration of NAIs inhibited the growth of bacteria. An early study showed that NAIs caused a significant amount of biological decay of the bacterium *Serratia marcescens* [95]. Exposure to NAIs showed inactivation or growth inhibition of the bacteria *E. coli*, *Candida albicans*, *Staphylococcus aureus*, *P. fluorescens* [96,97,98,99,100] and has a lethal effect on starved *Pseudomonas veronii* cells [101]. NAIs prevented 60% of tuberculosis (TB) infection and 51% of TB disease [102]. Except for the inhibition effect of NAIs on bacteria, reports also showed that NAIs inhibited the growth of fungi and viruses. For example, NAIs could inhibit the growth of *Penicillium notatum* [103]; the use of NAI generators reduced airborne transmission of Newcastle disease virus [104]. However, some controversial results have been reported on the growth inhibition of NAIs on microorganisms. For example, Fan et al. (2007) reported that NAIs had a very limited effect on *E. coli* that were inoculated on mung bean seed and apples [105]. In another report, seven bacterial species (*Staphylococcus aureus*, *Mycobacterium parafortuitum*, *Pseudomonas aeruginosa*, *Acinetobacter baumanii*, *Burkholderia cenocepacia*, *Bacillus subtilis*, and *Serratia marcescens*) were exposed to NAIs and only the growth *Mycobacterium parafortuitum* was inhibited [106].

Besides animals/humans and microorganisms, plants might also be affected by NAI exposure. After NAI treatment of *Avena sativa*, the fresh and dry weight were increased [107] and mean stem length and integral elongation were also increased [108]. Oxygen consumption was increased in barley seedlings after exposure to NAIs [109]. Plant height increased by 13–15% and dry weight increased by 18% under the growth environment with high concentrations of NAIs [110]. Lettuce plants exposed to NAIs showed vigorous growth with increased leaf area and fresh weight [111]. NAI treatment improved sprout growth and bacterial control during plant development [112]. NAIs have a positive effect on kale growth by improving fresh weight, macroelements and microelements [113]. However, no significant difference was observed between control and NAI-treated tobacco plants in nicotine and total alkaloid; the total nitrogen content in NAI-treated plants was slightly increased when compared with control plants [114].

## 7. Superoxide Involvement in the Biological Effects of Negative Air Ions

The oxygen molecule O_2_ is a biradical with two unpaired electrons in different orbitals and is capable of accepting up to 4 electrons [125]. Superoxide O_2_^•−^ is formed by accepting one electron. At least 4 oxidation states of oxygen have been identified including O_2_ (dioxygen), O_2_^+^ (dioxygen cation), O_2_^•^^−^ (superoxide) and O_2_^2−^ (peroxide dianion) [126]. The monovalent reduction of O_2_ gives birth to O_2_^•−^, which serves as both a radicle and a negative ion; thus, the radicle sign “^•^” is added [116]. Therefore, superoxide O_2_^•−^ is a kind of negative oxygen ions, which was documented by several reports [28,36,37,38,127]. Reports showed that superoxide ions are the major negative ions generated by natural atmosphere and the Lenard effect (waterfall) [37,38,128] and is more stable than other NAIs [25,127]. Superoxide O_2_^•−^ was also detected in those NAIs that were generated by stimulating plant roots using PEF [28].

An early study showed the involvement of superoxide in the bacterial killing of *Staphylococcus albus* [117]. As commented by Rosenthal and Ben-Hur (1980) [36], the result was supported by the result that the bacterial killing of *Staphylococcus albus* by NAIs was protected by adding superoxide dismutase. However, reports showed that many other factors might also lead to the observed lethal effect of NAIs on bacteria [36]. Inhaled gaseous superoxide might improve the antinociceptive effect of analgesic agents and relieved the pathological signs of Parkinson’s disease [2,129]. Goldstein et al. (1992) also reported that the biological activity of NAIs may be dependent on superoxide [128], but it must be emphasized that the biological effects of NAIs are due to not only superoxide, but also other activated oxygen species [127,130,131]. On the other hand, it also needs to be mentioned that gas-phase superoxide ions are different from liquid-phase superoxide [37]; thus, the biological effects of the former might also be different from those of the latter.

## 8. The Release of Negative Air Ions as an Efficient Way to Remove Fine Particulates

PM is the major pollutant during haze episodes. Evidence showed that PM, especially PM_2.5_, seriously affected human health. Based on the data from Lim et al. (2012), around 3.2 million people died in 2010 due to exposure to PM [132]. Annually, 2.1 million deaths occurred in the world due to the increasing levels of PM_2.5_ [133,134]. PM mainly led to diseases related to both respiratory and cardiovascular systems. Fine PM such as PM_1.0_ and PM_2.5_ penetrate deeply into the lung, irritate and corrode the alveolar wall and, as a result, affect lung functions [135] and might also result in lung cancer [136]. However, the deposition of particles in the respiratory tract is only enhanced for particles with many charges, which can only be achieved with an ionizer in an enclosed space. PM was also related to increased blood glucose, worse endothelial function, and incident cardiovascular disease events [137].

NAIs are unipolar ions and they will electrically charge PM, which will be deposited/precipitated more rapidly than uncharged ones as charged PM can attach to nearby surfaces, attach to each other and settle faster, or sink faster under the gravity [138,139,140,141,142] (available online: https://www.epa.gov/indoor-air-quality-iaq/guide-air-cleaners-home).

Tanaka et al. (1996) reported that NAIs reduced respirable and inhalable dust counts by 46% [143]. PM concentration was reduced by up to two orders of magnitude after 2 hours of treatment by NAI generator in an unoccupied office room (50 m^3^) [144]. Many other similar reports showed the high efficiency of NAIs in PM removal (Table 3; [138,140,145,146,147,148,149,150,151,152]). Evidence showed that many factors might affect PM reducing efficiency by NAIs (Table 3; [34,139,150,153]). For example, PM removal rate was related to particle concentrations, particle sizes, and the ventilation conditions and a model was raised to compute their relationship [139]. Studies also showed that wall surface significantly affected PM reducing efficiency [34]. In the chamber with the wood and polyvinyl chloride (PVC) wall surfaces, the NAI could remove PM particles substantially more effectively when compared with other wall materials such as wallpaper, stainless steel, and cement paint. One of the obvious disadvantages of ionizers is to emit ozone [154], which is a powerful oxidant and may seriously harm our health by long-term and/or high-dose exposure. Studies showed that many NAI generators including well-branded ionizers release ozone (Table 3; [141,145,155,156,157]). Another side effect is that the continuous emission of NAIs into an enclosed environment may lead to charge accumulation on insulating surfaces and then may cause electrostatic problems, especially when the humidity is low [138].

## 9. Prospects and Future Works on Plant-Based NAI Generator

As mentioned above, plants could release a huge amount of negative air ions under PEF stimulation. This is an interesting topic and many more works should be carried out to figure out the mechanisms for plants to help generate NAIs. Evidence showed that light intensity significantly increased the plant-based NAI release by PEF stimulation [28]. During PEF stimulation, more leaf stomata opened wider which might contribute to a higher level of NAI release [28]. Generally, limited data are available on how PEF stimulation helps plants to generate very high concentrations of NAIs. No data were reported on what happens during PEF stimulation and how the stimulation may regulate the profile of gene expression. On the other hand, no reports were available on the composition of these NAIs released through stimulating plants by PEF treatment and their biological effects. Furthermore, only limited data were available on the application of these plant-based NAIs on reducing PM (available online: https://patents.google.com/patent/CN203313745U/en?oq=CN+203313745+U). In a word, studies on the mechanism of plant-based release of NAIs by PEF stimulation are still under way. More efforts should be carried out on the comparison between these plant-based NAIs and other sources of NAIs and their difference in biological effects and PM reduction.

## 10. Conclusions

**Figure 3:**
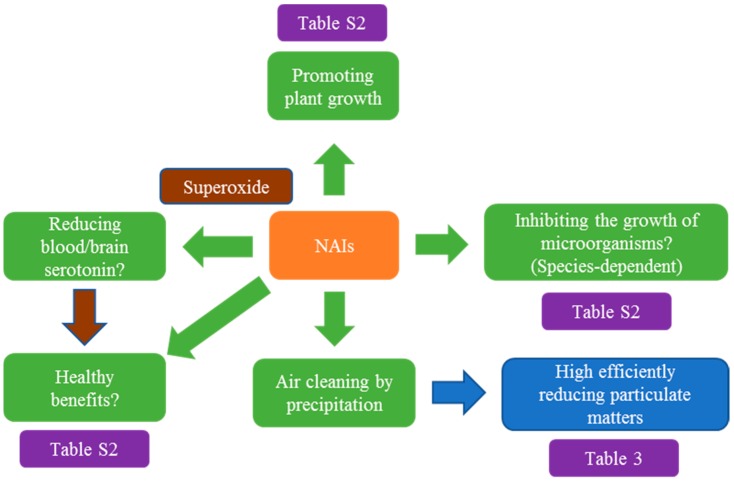
A summary of the benefits of negative air ions on organisms and air cleaning.

Some studies have suggested that NAIs had multiple health benefits on humans/animals, might inhibit the growth and/or kill some of microorganisms and promote plant development (Figure 3), but some of the results need to be further verified, some references might overestimate its benefits and no consistent or reliable evidence in therapeutic effects were achieved. However, to our knowledge, no data showed the harmful effects of NAIs on humans/animals. Superoxide ions are key members of NAIs and have been involved in the biological effects of NAIs by regulating the serotonin level and other biological actions but some reports showed no significant effect of NAIs on the concentration or turnover of serotonin. On the other hand, evidence showed that NAIs could high-efficiently remove PM including ultrafine PM (Figure 3), providing an alternative way to clean indoor air especially during haze episodes. We have also reviewed the plant-based NAI generation system by PEF stimulation. More efforts should be input into this system to further improve it and to use it as a high-efficient NAI generator and PM removal system.

## Figures and Tables

**Table 1:** NAIs released by different plants under natural growth conditions without pulse electric field (PEF) stimulation.

Treatment	Distance between Ion Counter and Plant	NAI Concentration (ions/cm^3^)	Reference
Soil pot without plants	70 cm	135 ± 70 ^#^	[28]
Soil pot with plants	70 cm	133 ± 45 ^#^	[28]
Soil pot without plants	≤32.5 cm *	35	[30]
Soil pot with plants			
*M. adansonii var. laniata*	≤32.5 cm *	36	[30]
*C. asiaticum var. sinicum*	≤32.5 cm *	68	[30]
*O. bodinieri*	≤32.5 cm *	56	[30]
*A. macrorrhizos*	≤32.5 cm *	66	[30]
*A. densiflorus*	≤32.5 cm *	55	[30]
*D. ensifolia*	≤32.5 cm *	40	[30]
*N. tazetta var. chinensis*	≤32.5 cm *	73	[30]
*H. fulva*	≤32.5 cm *	55	[30]
*Z. carinata*	≤32.5 cm *	72	[30]
*D. cochinchinensis*	≤32.5 cm *	43	[30]

* Specific distance may be related to plant size, please refer to the cited paper for more details. # Average values for all used plant species under 60 min of experiment.

**Table 2:** NAIs released by different plants under different treatments with PEF stimulation.

Plant Species/Variety/Treatment	Distance between Ion Counter and Plant	Output Voltage	NAI Concentration (ions/cm^3^)	Reference
Soil pot without plants	≤32.5 cm *	25 kv *	131 ± 4	[30]
Soil pot without plants	70 cm	20 kv	4000 ± 400	[28]
*Aloe arborescens*	70 cm	25 kv	280,000 ± 41,000	[28]
*Haworthia rasalata*	70 cm	25 kv	41,000 ± 8000	[28]
*Chlorophytum comosum*	70 cm	25 kv	95,000 ± 12,000	[28]
*Opuntia brunnescens*	70 cm	25 kv	53,000 ± 13,000	[28]
*Zephyranthes carinata*	≤32.5 cm *	15 kv	1,791,067 ± 27,243	[30]
*Zephyranthes carinata*	≤32.5 cm *	20 kv	3,593,489 ± 358,104	[30]
*Crinum asiaticum*	≤32.5 cm *	15 kv	201,000 ± 23,798	[30]
*Crinum asiaticum*	≤32.5 cm *	20 kv	59,475 ± 839	[30]
*Narcissus tazetta*	≤32.5 cm *	15 kv	162 ± 28	[30]
*Narcissus tazetta*	≤32.5 cm *	20 kv	315 ± 33	[30]
*Ophiopogon japonicus*	Near the grass	37.6 kv	2,000,000 ± 300,000	[29]
*L. Muttiflorum*	Near the grass	37.3 kv	500,000 ± 60,000	[29]
*Zoysia spp*	Near the grass	38 kv	200,000 ± 30,000	[29]
*Poa pratensis*	Near the grass	38.6 kv	200,000 ± 30,000	[29]
*Zephyranthes candida*	Near the grass	42.8 kv	100,000 ± 23,000	[29]
*Apocynum venetum*	Near the grass	30.7 kv	80,000 ± 9000	[29]

* Specific distance may be related to plant size, please refer to the cited paper for more details. kv: kilovoltage.

**Table 3:** The effect of NAIs on particulate matter (PM) removal.

Item	Description	Reference
PM removal efficiency	NAIs reduced respirable and inhalable dust counts by 46%.	[143]
	PM concentration was reduced by up to two orders of magnitude after 2 h of treatment by NAI generator d in a 50 m^3^ unoccupied office room.	[144]
	From around 800 µg/m^3^ to 50 µg/m^3^ in a chamber (2 m × 2 m × 1.6 m)	[145]
	Ionizer is efficient in reducing fine PM	[146]
	More than 80% 0.1 or 1 mm particles were removed within 1 h	[138]
	The removal efficiency under high concentration of NAIs reached about 50% after 15 min and almost 100% after 1.5 h.	[140]
	In a glass chamber (60 cm × 30 cm × 40 cm), 93% to 97% of the particles from fog or smoke was removed by NAIs within 6 min.	[147]
	Around 95% of respirable particles from indoor air can be removed	[148]
	The particle removal efficiency increases with enhanced ion emission rate and the duration of emission.	[149]
	NAIs can be used to eliminate cigarette smoke	[150]
	Air ionizers are efficient even in a ventilated room with 132 m^3^ and can be used for removing ultrafine particles.	[151]
	Field tests conducted in Shanghai showed stable PM_2.5_ purification efficiency of 99.99% at high releasing amounts of negative ions (RANIs), in the event of haze.	[152]
Factors affecting PM reduction	PM removal rate is related to particle concentrations, particle sizes, and the ventilation rate and a model is raised to compute the dependence.	[139]
	The NAI could remove particles from the wood and polyvinyl chloride (PVC) wall surfaces substantially more effectively than from other wall materials such as wallpaper, stainless steel, and cement paint.	[34]
	PM reducing efficiency was related to the heights and distances from the source of NAIs. The highest efficiency of PM_10_ removal was achieved when the distance between smoking cigarettes and ionizer was 3 m and the air humidity was 39%.	[150]
	PM diameter significantly affects the deposition of particles and wall roughness is a key beneficial factor to particle deposition driven by ionizer	[153]
Drawback	Ozone was produced by corona discharge of air	[154]
	A total of 5 out of the 27 ionizers were found to emit ozone	[145]
	The high voltage used for ion generation produces Ozone above the threshold voltage of 16,000 volts in the tested system.	[155]
	The ozone levels were increased by at least 3 times after using a leading commercially available ionic air cleaner	[141]
	Portable air cleaners can both remove and generate pollutants indoors due to ozone release	[156]
	Many NAI generators emitted ozone	[157]

## Supplementary Materials

Supplementary materials can be found at http://www.mdpi.com/1422-0067/19/10/2966/s1..

## Abbreviations

NAI	Negative air ion
PM	Particulate matter
PEF	Pulsed electric field
UV	Ultraviolet
SA	Salicylic acid
MV	Methyl viologen
KV	Kilovoltage
RANIs	Releasing amounts of negative ions
PVC	Polyvinyl chloride
