# Supplementary material for: Negative Air Ions and Their Effects on Human Health and Air Quality Improvement
Source: Int J Mol Sci. 2018 Sep 28;19(10):2966. doi: 10.3390/ijms19102966 (PMC6213340; doi:10.3390/ijms19102966)
Supplement: Supplementary file 1 [file ijms-19-02966-s001.zip › ijms-350701 Supplementary material for final/ijms-350701 Supplementary Experiment.pdf]

Negative Air Ions and Their Effects on Human Health and Air Quality improvement

Supplementary Experiment: The Effect of UV Lighting on Negative Air Ion Generation

1. Introduction

Negative air ions (NAIs) are negatively charged molecules or atoms. Many factors may contribute to the generation of NAIs, which include radiant or cosmic rays in the atmosphere, sunlight, natural and artificial corona discharge, shearing water and growing plants [1–11]. Not all wavelength of sunlight can be used to generate NAIs. Reports showed that ultraviolet (UV) can be used to directly ionize air molecules to generate NAIs [12,13]. Evidence showed that in the above 60 km altitude of atmosphere, the dominant NAIs were generated by UV-mediated ionization [12]. However, in the lower atmosphere, only low dose of UV rays is available and as a result, ionization by UV radiation is not a major contributor of NAIs in this atmosphere layer [12]. Thus, data on the effects of UV rays on NAI generation were mainly from the studies on atmosphere, where UV rays were naturally emitted. Little systematical study was carried out on the effect of artificial UV lighting on NAI generation. Here, we carried out an experiment to investigate the contribution of UV lighting to the generation of NAIs by comparing with the case under normal lighting conditions.

2. Materials and Methods

A man-made chamber with dimensions of 80 cm length, 80 cm width and 80 cm height was used for this experiment. The chamber was made from 6 mm thick clear Perspex for all six sides. A 30 watts of UV light was provided by Safer Electric Ltd., Singapore, which emits UV-C light with wavelength of 100–280 nm. The UV light tube was installed on the top side of the chamber. The air ion counter with the model DLY-4G-232 was purchased from Kilter Electronic Institute Co., Ltd. (Zhangzhou, Fujian Province, China), which can measure NAI concentration from 0 to 1.999×10^9 ion/cm^3. The air ion counter was placed into the centre of the bottom side of the growth chamber. The air ion counter continuously monitors the NAI concentration in the chamber and generates one read per second, which are recorded in the connected computer. The control experiment was carried out in the same chamber with a 30-watt of normal light instead of UV light. A total of 3 replicates were carried out under either control or UV lighting conditions. The NAI concentrations were monitored continuously for 1 hour for each treatment. For all three replicates or each replicate, a nonparametric test of two independent samples (i.e., the two-tailed Mann-Whitney U test, n = 10803 in CK and n = 10803 in UV lighting for three replicates; n = 3601 in CK and n = 3601 in UV lighting for each replicate) was performed with the Type I error (α value) set to 0.05. The Mann-Whitney U test were performed using the software package SPSS Statistics version 22 (SPSS Inc., Chicago, Illinois, USA).

3. Results and Discussion

To survey the contribution of artificial UV lighting to NAI generation, we set up a control experiment (CK), where only a 30-watt of normal light was installed in the chamber. The average NAI concentration under normal lighting conditions was 344 ions/cm^3 (Figure 1A). During the one-hour testing period, no significant difference in the NAI concentration has been observed among three different replicates (Figure 1B–C). However, under UV lighting conditions, the average NAI concentration was increased to 825 ions/cm^3 (Figure 1A). The Mann-Whitney U test analysis by all three replicates showed that UV lighting significantly promoted NAI generation (p < 0.00001). Further observation showed that there were peaks of NAI generation within 8 minutes after UV lighting (Figure 1B–D; Table S1). After the peaks, NAI concentration was kept at a relatively stable value but was still

higher than the control. For each replicate, a nonparametric two-tailed Mann-Whitney U test was carried out as described in Materials and methods and the statistical analysis showed that NAI concentrations under UV lighting conditions in all three replicates were significantly higher than those under normal lighting conditions (CK) with p value less than 0.00001. The analysis further confirmed the promoting effect of UV lighting on NAI generation.

Our experiment shows that UV lights could be used to generate NAIs. However, only low amount concentrations of NAIs were generated under our UV light conditions. Reports showed that the threshed value of negative oxygen ion concentration for fresh air should be more than 1000 ions/cm^3 and the concentration should be higher for boosting the human immune system ([14] and references therein). The average NAI concentration under UV lighting is 825 ions/cm^3, less than the threshed value (Figure 1A) and thus, UV lighting under our experiment is not an ideal method to generate NAIs. UV light has been widely used in hospitals for various applications [15]. What we need to emphasize is that the experiment in Figure 1 is just to verify the contribution of UV light to the generation of NAIs. We used man-made small chamber but not a space to mimic hospital conditions as our experiment was not to verify the possible application of UV lighting for generating NAIs in hospitals. Although UV lighting significantly contributes to NAI generation, it is not a good ideal for us to use UV light to generate NAIs in hospitals.

References

1. Hoppel, W.A.; Anderson, R.V.; Willet, J.C. *Atmospheric electricity in the planetary boundary layer, The Earth's Electrical Environment*; NAS Press: Washington, D.C., USA, 1986; pp. 195-205.
2. Ermakov, V.I.; Bazilevskaya, G.A.; Potrevsky, P.E.; Stozhkov, Y.I. Ion balance equation in the atmosphere. *J. Geophys. Res.* **1997**, *102*, 23413–23419, doi:10.1029/97JD01388.
3. Borra, J.P.; Roos, R.A.; Renard, D.; Lazar, H.; Goldman, A.; Goldman, M. Electrical and chemical consequences of point discharges in a forest during a mist and a thunderstorm. *J. Phys. D: Appl. Phys.* **1997**, *30*, 84–93, doi:10.1029/2002JD002345.
4. Aubrecht, L.; Stanek, Z.; Koller, J. Corona discharge on coniferous trees—Spruce and pine. *Europhys. Lett.* **2001**, *53*, 304–390, doi:10.1209/epl/i2001-00153-2.
5. Murr, L.E. Plant growth response in a simulated electric field-environment. *Nature* **1963**, *200*, 490-491, doi:10.1038/200490b0.
6. Bachman, C.H.; Hademenos, D.G. Ozone and air ions accompanying biological applications of electric fields. *J. Atmos. Terr. Phys.* **1971**, *33*, 497–505, doi:10.1016/0021-9169(71)90153-X.
7. Krueger, A.P.; Strubbe, A.E.; Yost, M.G.; Reed, E.J. Electric fields, small air ions and biological effects. *Int. J. Biometeorol.* **1978**, *22*, 202–212, doi:10.1007/BF01555399.
8. Hart, F.X.; Schottenfeld, R.S. Evaporation and plant damage in electric fields. *Int. J. Biometeor.* **1979**, *23*, 63–68, doi:10.1007/BF01553377.
9. Hart, F.X.; Mudano, M.J.; Atchley, A.A. Plant damage produced by the passage of low level direct current. II. Further observations and possible mechanisms. *Int. J. Biometeor.* **1981**, *25*, 151–159, doi:10.1007/BF02184463.
10. Lenard, P. Über die Electrizität der Wasserfälle. *Ann. Phys.* **1892**, *46*, 584–636.
11. Iwama, H. Negative air ions created by water shearing improve erythrocyte deformability and aerobic metabolism. *Indoor Air* **2004**, *14*, 293–297, doi:10.1111/j.1600-0668.2004.00254.x.
12. Harrison, R.G.; Carslaw, K.S. Ion-aerosol-cloud processes in the lower atmosphere. *Rev. Geophys.* **2003**, *41*, doi:10.1029/2002RG000114.
13. Yates, A.; Gray, F.B.; Misiaszek, J.I.; Wolman, W. Air ions: past problems and future directions. *Environ Int.* **1986**, *12*, 99–108, doi:10.1016/0160-4120(86)90019-X.
14. Lazzerini, F.T.; Orlando, M.T.; De Prá, W. Progress of negative air ions in health tourism environments applications. *Bol. Soc. Esp. Hidrol. Méd.* **2018**, *33*, 27–46, doi:10.23853/bsehm.2018.0450.
15. Ahmad, S.I.; Christensen, L.; Baron, E. History of UV Lamps, Types, and Their Applications. *Adv. Exp. Med. Biol.* **2017**, *996*, 3–11, doi:10.1007/978-3-319-56017-5_1.

© 2018 by the authors. Submitted for possible open access publication under the terms and conditions of the Creative Commons Attribution (CC BY) license (http://creativecommons.org/licenses/by/4.0/).
