# Supplementary material for: Negative Air Ions and Their Effects on Human Health and Air Quality Improvement
Source: Int J Mol Sci. 2018 Sep 28;19(10):2966. doi: 10.3390/ijms19102966 (PMC6213340; doi:10.3390/ijms19102966)
Supplement: Supplementary file 1 [file ijms-19-02966-s001.zip › ijms-350701 Supplementary material for final/ijms-350701 Table S2.docx]

**Negative Air Ions and Their Effects on Human Health and Air Quality improvement**

**Table S2.** Reports of NAIs on organisms

| **Organisms** | **Description** | **References** |
| --- | --- | --- |
| Humans and animals | Significant increase in performance of all tested tasks exposed to NAIs | [1] |
|  | NAIs affect cerebral cortex, serotonin, and cyclic nucleotides | [2] |
|  | NAIs from tourmaline ionizer decreased the blood pressure in rats | [3] |
|  | NAIs lower blood pressure, serotonin, and dopamine | [4] |
|  | NAIs attenuate blood lactate concentrations | [5] |
|  | NAIs improve erythrocyte deformability and aerobic metabolism | [6] |
|  | NAIs are effective for treatment of chronic depression | [7] |
|  | NAIs activate natural killer (NK) cell and inhibit carcinogenesis in mice | [8] |
|  | Inhaling of NAIs by rats activated the secretion of goblet cells | [9] |
|  | NAIs does not play an appreciable role in respiratory function | [10] |
|  | NAI treatment for mood disorders is in general effective | [11] |
|  | Exposing humans to NAIs reduced blood pressure | [12] |
|  | Slightly activating and cognitive performance enhancing effects | [13] |
|  | Exposure to NAIs alleviates symptoms of seasonal affective disorder | [14] |
|  | No effect of NAIs on the concentration or turnover of serotonin in rats | [15] |
|  | No effect of NAIs on lowering blood pressure | [16] |
|  | No effect of NAIs on heart rate | [17] |
|  | No effect of NAIs on mental health | [18,19] |
|  | A systematic review suggested no consistent or reliable effects of NAIs on cardiovascular and respiratory system as well as on mental health | [20] |
| Bacteria | Significant amount of biological decay of bacterium Serratia marcescens | [21] |
|  | Superoxide involvement in the bactericidal effects on *Staphylococcus albus* | [22] |
|  | Ionizer can be used to reduce the microbial air pollution in a dental clinic | [23] |
|  | NAIs resulted in a significant growth inhibition of *Candida albicans* | [24] |
|  | High levels of NAIs directly killed *Salmonella Enteritidis* | [25] |
|  | Exposure to NAIs produced significant reductions in colony number | [26] |
|  | The synergism of ozone with NAIs may suppress certain bacteria | [27] |
|  | Exposure to NAIs has a lethal effect on starved *Pseudomonas veronii* cells. | [28] |
|  | Ionisers may have a role in the prevention of Acinetobacter infections. | [29] |
|  | NAIs have a very limited effect on *E. coli* on mung bean seed and apples | [30] |
|  | The bactericidal action due to NAIs may have been overestimated. | [31] |
|  | NAIs inhibited the growth of *E. coli* and *P. fluorescens* | [32] |
|  | NAIs prevented 60% of tuberculosis (TB) infection and 51% of TB disease | [33] |
|  | Ionizer inhibited airborne bacteria | [34] |
|  | The anti-*Pseudomonas fluorescens* effect of the essential oil vapours can be significantly enhanced by the addition of NAI. | [35] |
|  | NAIs showed inactivation of the bacteria *E. coli* | [36] |
|  | NAIs reduced *E. coli* and *Staphylococcus aureus* by more than 97% | [37] |
|  | Disinfection of *Serratia marcescens* and *Staphylococcus epidermidis* | [38] |
| Fungus | NAIs inhibit the growth of Penicillium notatum | [39] |
| Virus | Ionizer reduced airborne transmission of the Roakin strain of Newcastle disease virus (NDV) at an average of 6.6% to 27.7% | [40] |
| Plants | The fresh and dry weight of *Avena sativa* increased after NAI treatment | [41] |
|  | NAIs promoted the growth of *Avena sativa* seedlings | [41,42] |
|  | Oxygen consumption was increased in barley after exposure to NAIs | [43] |
|  | NAIs increased plant height by 13-15% and dry weight by 18% | [44] |
|  | NAIs slightly increased total nitrogen content | [45] |
|  | Lettuce plants exposed to NAIs increased leaf area and fresh weight | [46] |
|  | NAIs improved sprout growth and bacterial control | [47] |
|  | NAIs improved fresh weight, macroelements and microelements in kale | [48] |

References:

1. Hawkins, L.H.; Barker, T. Air ions and human performance. *Ergonomics* **1978**, *21*, 273–278, doi:10.1080/00140137808931724.
2. Diamond, M.C.; Connor, J.R.Jr.; Orenberg, E.K.; Bissell, M.; Yost, M.; Krueger, A. Environmental influences on serotonin and cyclic nucleotides in rat cerebral cortex. *Science* **1980**, *210*, 652–654, doi:10.1126/science.6254145.
3. Ju, K.; Kubo, T. Power spectral analysis of autonomic nervous activity in spontaneously hypertensive rats. *Biomed. Sci. Instrum.* **1997**, *33*, 338–343.
4. Ryushi, T.; Kita, I.; Sakurai, T.; Yasumatsu, M.; Isokawa, M.; Aihara, Y.; Hama, K. The effect of exposure to negative air ions on the recovery of physiological responses after moderate endurance exercise. *Int. J. Biometeorol.* **1998**, *41*, 132–136, doi:10.1007/s004840050066.
5. Iwama, H.; Ohmizo, H.; Furuta, S.; Ohmori, S.; Watanabe, K.; Kaneko, T.; Tsutsumi, K. Inspired superoxide anions attenuate blood lactate concentrations in postoperative patients. *Crit. Care Med.* **2002**, *30*, 1246–1249.
6. Iwama, H. Negative air ions created by water shearing improve erythrocyte deformability and aerobic metabolism. *Indoor Air* **2004**, *14*, 293–297, doi:10.1111/j.1600-0668.2004.00254.x.
7. Goel, N.; Terman, M.; Terman, J.S.; Macchi, M.M.; Stewart, J.W. Controlled trial of bright light and negative air ions for chronic depression. *Psychol. Med.* **2005**, *35*, 945–955, doi:10.1017/S0033291705005027.
8. Yamada, R.; Yanoma, S.; Akaike, M.; Tsuburaya, A.; Sugimasa, Y.; Takemiya, S.; Motohashi, H.; Rino, Y.; Takanashi, Y.; Imada, T. Water-generated negative air ions activate NK cell and inhibit carcinogenesis in mice. *Cancer Lett.* **2006**, *239*, 190–197, doi:10.1016/j.canlet.2005.08.002.
9. Sirota, T.V.; Safronova, V.G.; Amelina, A.G.; Mal'tseva, V.N.; Avkhacheeva, N.V.; Sofin, A.D.; Ianin, V.A.; Mubarakshina, E.K.; Romanova, L.K.; Novoselov, VI. Effect of negative air ions on respiratory organs and blood. *Biofizika* **2008**, *53*, 886–893, doi:10.1134/S0006350908050242.
10. Alexander, D.D.; Bailey, W.H.; Perez, V.; Mitchell, M.E.; Su, S. Air ions and respiratory function outcomes: A comprehensive review. *J. Negat. Results Biomed.* **2013**, *12*, 14, doi:10.1186/1477-5751-12-14.
11. Pino, O.; Ragione, F.L. There’s something in the air: Empirical evidence for the effects of negative air ions (NAI) on psychophysiological state and performance. *Res. Psychol. Behav Sci.* **2013**, *1*, 48–53.
12. Wiszniewski, A.; Suchanowski, A.; Wielgomas, B. Effects of Air-Ions on human circulatory indicators. *Pol. J. Environ. Stud.* **2014**, *23*, 521–531.
13. Wallner, P.; Kundi, M.; Panny, M.; Tappler, P.; Hutter, H.P. Exposure to air ions in indoor environments: Experimental study with healthy adults. *Int. J. Environ. Res. Public Health.* **2015**, *12*, 14301–14311, doi:10.3390/ijerph121114301.
14. Bowers, B.; Flory, R.; Ametepe, J.; Staley, L.; Patrick, A.; Carrington, H. Controlled trial evaluation of exposure duration to negative air ions for the treatment of seasonal affective disorder. *Psychiatry Res.* **2018**, *259*, 7–14, doi:10.1016/j.psychres.2017.08.040.
15. Bailey, W.H.; Charry, J.M. Acute exposure of rats to air ions: Effects on the regional concentration and utilization of serotonin in brain. *Bioelectromagnetics.* **1987**, *8*, 173–181.
16. Watanabe, I.; Noro, H.; Ohtsuka, Y.; Mano, Y.; Agishi, Y. Physical effects of negative air ions in a wet sauna. *Int. J. Biometeorol.* **1997**, *40*, 107–112, doi:10.1007/s004840050028.
17. McDonald, R.D.; Bachman, C.H.; Lorenz, P.J. Some physiological effects of air ion treatment without ion inhalation. *Int. J Biometeorol.* **1965**, *9*, 141–147, doi:10.1007/bf02188469.
18. Hedge, A.; Collis, M.D. Do negative air ions affect human mood and performance? *Ann. Occup. Hyg.* 1987, 31, 285–290.
19. Reilly, T.; Stevenson, I.C. An investigation of the effects of negative air ions on responses to submaximal exercise at different times of day. *J. Hum. Ergol. (Tokyo).* **1993**, *22*, 1–9.
20. Bailey, W.H.; Williams, A.L.; Leonhard, M.J. Exposure of laboratory animals to small air ions: A systematic review of biological and behavioral studies. *BioMed.* *Eng. OnLine.* **2018**, *17*, 72, doi:10.1186/s12938-018-0499-z.
21. Phillips, G.; Harris, G.J.; Jones, M.W. Effects of air ions on bacterial aerosols. *Int. J. Biometeorol.* **1964**, *8*, 27–37, doi:10.1007/BF02186925.
22. Kellogg, E.W.; Yost, M.G.; Barthakur, N.; Kreuger, A.P. Superoxide involvement in the bactericidal effects of negative air ions on *Staphylococcus albus*. *Nature* **1979**, *281*, 400–401, doi:10.1038/281400a0.
23. Gabbay, J.; Bergerson, O.; Levi, N.; Brenner, S.; Eli, I. Effect of ionization on microbial air pollution in the dental clinic. *Environ. Res.* **1990**, *52*, 99–106, doi:10.1016/S0013-9351(05)80154-9.
24. Shargawi, J.M.; Theaker, E.D.; Drucker, D.B.; MacFarlane, T.; Duxbury, A.J. Sensitivity of *Candida albicans* to negative air ion streams. *J. Appl. Microbiol.* **1999**, *87*, 889–897, doi:10.1046/j.1365-2672.1999.00944.x.
25. Seo, K.H.; Mitchell, B.W.; Holt, P.S.; Gast, R.K. Bactericidal effects of negative air ions on airborne and surface Salmonella enteritidis from an artificially generated aerosol. *J. Food Prot.* **2001**, *64*, 113–116, doi:10.4315/0362-028X-64.1.113.
26. Noyce, J.O.; Hughes, J.F. Bactericidal effects of negative and positive ions generated in nitrogen on *Escherichia coli*. *J. Electrostatics* **2002**, *54*, 179–187, doi:10.1016/S0304-3886(01)00179-6.
27. Fan, L.; Song, J.; Hildebrand, P.D.; Forney, C.F. Interaction of ozone and negative air ions to control micro-organisms. *J. Appl. Microbiol.* **2002**, *93*, 144–148, doi:10.1046/j.1365-2672.2002.01683.x.
28. Noyce, J.O.; Hughes, J.F. Bactericidal effects of negative and positive ions generated in nitrogen on starved *Pseudomonas veronii*. *J. Electrostatics* **2003**, *57*, 49–58, doi:10.1016/S0304-3886(02)00110-9.
29. Kerr, K.G.; Beggs, C.B.; Dean, S.G.; Thornton, J.; Donnelly, J.K.; Todd, N.J.; Sleigh, P.A.; Qureshi, A.; Taylor, C.C. Air ionisation and colonization / infection with methicillin-resistant *Staphylococcus aureus* and *Acinetobacter* species in an intensive care unit. *Intensive Care Med.* **2006**, *32*, 315–317, doi:10.1007/s00134-005-0002-8.
30. Fan, X.; Fett, W.F.; Mitchell, B.W. Effect of negative air ions on *Escherichia coli* ATCC 25922 inoculated onto mung bean seed and apple fruit. *J. Food Prot.* **2007**, *70*, 204–208, doi:10.4315/0362-028X-70.1.204.
31. Fletcher, L.A.; Gaunt, L.F.; Beggs, C.B.; Shepherd, S.J.; Sleigh, P.A.; Noakes, C.J.; Kerr, K.G. Bactericidal action of positive and negative ions in air. *BMC Microbiol.* **2007**, *7*, 32, doi:10.1186/1471-2180-7-32.
32. Tyagi, A.K.; Nirala, B.K.; Malik, A.; Singh, K. The effect of negative air ion exposure on Escherichia coli and Pseudomonas fluorescens. *J. Environ. Sci. Health A Tox. Hazard Subst. Environ. Eng.* **2008**, *43*, 694–699, doi:10.1080/10934520801959831.
33. Escombe, A.R.; Moore, D.A.; Gilman, R.H.; Navincopa, M.; Ticona, E.; Mitchell, B.; Noakes, C.; Martínez, C.; Sheen, P.; Ramirez, R.; et alUpper-room ultraviolet light and negative air ionization to prevent tuberculosis transmission. *PLoS Med.* **2009**, *6*, e43, doi:10.1371/journal.pmed.1000043.
34. Kampmann, Y.; Klingshirn, A.; Kloft, K.; Kreyenschmidt, J. The application of ionizers in domestic refrigerators for reduction in airborne and surface bacteria. *J. Appl. Microbiol.* **2009**, *107*, 1789–1798, doi:10.1111/j.1365-2672.2009.04359.x.
35. Tyagi, A.K.; Malik, A. Antimicrobial action of essential oil vapours and negative air ions against Pseudomonas fluorescens. *Int. J. Food Microbiol.* **2010**, *143*, 205–210, doi:10.1016/j.ijfoodmicro.2010.08.023.
36. Dobrynin, D.; Friedman, G.; Fridman, A.; Starikovskiy, A. Inactivation of bacteria using DC corona discharge: Role of ions and humidity. New J. Phys. **2011**, pii: 103033, doi:10.1088/1367-2630/13/10/103033.
37. Timoshkin, I.V.; Maclean, M.; Wilson, M.P.; Given, M.J.; MacGregor, S.J.; Wang, T.; Anderson, J.G. Bactericidal effect of corona discharges in atmospheric air. *IEEE T. Plasma Sci.* **2012**, *40*, 2322–2333, doi:10.1109/TPS.2012.2193621.
38. Zhou, P.; Yang, Y.; Huang, G.; Lai, A.C.K. Numerical and experimental study on airborne disinfection by negative ions in air duct flow. *Build. Environ.* **2018**, *127*, 204–210, doi:10.1016/j.buildenv.2017.11.006.
39. Pratt, R.; Barnard, R.W. Some effects of ionized air on *Penicillium notatum*. *J. Am. Pharm. Assoc. Sci.* **1960**, *49*, 643–646, doi:10.1002/jps.3030491004.
40. Mitchell, B.W.; King, D.J. Effect of negative air ionization on airborne transmission of newcastle disease virus. *Avian. Dis.* **1994**, *38*, 725–732, doi:10.2307/1592107.
41. Krueger, A.P.; Kotaka, S.; Andriese, P.C. Studies on the Effects of Gaseous Ions on Plant Growth I. The influence of positive and negative air ions on the growth of *Avena sativa*. *J. Gen Physiol.* **1962**, *45*, 879–895, doi:10.1085/jgp.45.5.879.
42. Krueger, A.P.; Kotaka, S.; Andriese, P.C. Some observations on the physiological effects of gaseous ions. *Int. J. Biometeorol.* **1962**, *6*, 33–48, doi:10.1007/BF02187011.
43. Kotaka, S.; Krueger, A.P.; Andriese, P.C.; Nishizawa, K.; Ohuchi, T.; Takenobu, M.; Kozure, Y. Air ion effects on the oxygen consumption of barley seedlings. *Nature* **1965**, *208*, 1112–1113, doi:10.1038/2081112a0.
44. Elkiey, T.M.; Bhartendu, S.; Barthakur, N. Air ion effect on respiration and photosynthesis of barley and *Antirrhinum majus*. *Int. J. Biometeorol.* **1985**, *29*, 285–292, doi:10.1007/BF02189659.
45. Barthakur, N.N.; Arnold, N.P. Growth and certain chemical constituents of tobacco plants exposed to air ions. *Int. J. Biometeorol.* **1988**, *32*, 78–80, doi:10.1007/BF01044897.
46. Song, M.J.; Kang, T.H.; Han, C.S.; Oh, M.M. Air anions enhance lettuce growth in plant factories. *Hortic. Environ. Biotechnol.* ***2014***, *55*, 293–298, doi:10.1007/s13580-014-1016-3.
47. Song, M.J.; Jeon, Y.M.; Oh, M.M. Growth of and bacterial counts on several edible sprouts exposed to spray ionization. *Protected Hort. Plant Fac.* **2015**, *24*, 45–50, doi:10.12791/KSBEC.2015.24.1.045.
48. Lee, S.R.; Kang, T.H.; Han, C.S.; Oh, M.M. Air anions improve growth and mineral content of kale in plant factories. *Hortic. Environ. Biotechnol.* **2015**, *56*, 462–471, doi:10.1007/s13580-015-0035-z.
