# Supplementary material for: Negative Air Ions and Their Effects on Human Health and Air Quality Improvement
Source: Int J Mol Sci. 2018 Sep 28;19(10):2966. doi: 10.3390/ijms19102966 (PMC6213340; doi:10.3390/ijms19102966)
Supplement: Supplementary file 1 [file ijms-19-02966-s001.zip › ijms-350701 Supplementary material for final/ijms-350701 Table S2.pdf]

Negative Air Ions and Their Effects on Human Health and Air Quality improvement

Table S2. Reports of NAIs on organisms

Organisms	Description	References
Humans and animals	Significant increase in performance of all tested tasks exposed to NAIs	[1]
	NAIs affect cerebral cortex, serotonin, and cyclic nucleotides	[2]
	NAIs from tourmaline ionizer decreased the blood pressure in rats	[3]
	NAIs lower blood pressure, serotonin, and dopamine	[4]
	NAIs attenuate blood lactate concentrations	[5]
	NAIs improve erythrocyte deformability and aerobic metabolism	[6]
	NAIs are effective for treatment of chronic depression	[7]
	NAIs activate natural killer (NK) cell and inhibit carcinogenesis in mice	[8]
	Inhaling of NAIs by rats activated the secretion of goblet cells	[9]
	NAIs does not play an appreciable role in respiratory function	[10]
	NAI treatment for mood disorders is in general effective	[11]
	Exposing humans to NAIs reduced blood pressure	[12]
	Slightly activating and cognitive performance enhancing effects	[13]
	Exposure to NAIs alleviates symptoms of seasonal affective disorder	[14]
	No effect of NAIs on the concentration or turnover of serotonin in rats	[15]
	No effect of NAIs on lowering blood pressure	[16]
	No effect of NAIs on heart rate	[17]
	No effect of NAIs on mental health	[18,19]
	A systematic review suggested no consistent or reliable effects of NAIs on cardiovascular and respiratory system as well as on mental health	[20]
Bacteria	Significant amount of biological decay of bacterium Serratia marcescens	[21]
	Superoxide involvement in the bactericidal effects on *Staphylococcus albus*	[22]
	Ionizer can be used to reduce the microbial air pollution in a dental clinic	[23]
	NAIs resulted in a significant growth inhibition of *Candida albicans*	[24]
	High levels of NAIs directly killed *Salmonella Enteritidis*	[25]
	Exposure to NAIs produced significant reductions in colony number	[26]
	The synergism of ozone with NAIs may suppress certain bacteria	[27]
	Exposure to NAIs has a lethal effect on starved *Pseudomonas veronii* cells.	[28]
	Ionisers may have a role in the prevention of Acinetobacter infections.	[29]
	NAIs have a very limited effect on *E. coli* on mung bean seed and apples	[30]
	The bactericidal action due to NAIs may have been overestimated.	[31]
	NAIs inhibited the growth of *E. coli* and *P. fluorescens*	[32]
	NAIs prevented 60% of tuberculosis (TB) infection and 51% of TB disease	[33]
	Ionizer inhibited airborne bacteria	[34]
	The anti-*Pseudomonas fluorescens* effect of the essential oil vapours can be significantly enhanced by the addition of NAI.	[35]
	NAIs showed inactivation of the bacteria *E. coli*	[36]
	NAIs reduced *E. coli* and *Staphylococcus aureus* by more than 97%	[37]
	Disinfection of *Serratia marcescens* and *Staphylococcus epidermidis*	[38]
Fungus	NAIs inhibit the growth of Penicillium notatum	[39]
Virus	Ionizer reduced airborne transmission of the Roakin strain of Newcastle disease virus (NDV) at an average of 6.6% to 27.7%	[40]
Plants	The fresh and dry weight of *Avena sativa* increased after NAI treatment	[41]
	NAIs promoted the growth of *Avena sativa* seedlings	[41,42]
	Oxygen consumption was increased in barley after exposure to NAIs	[43]
	NAIs increased plant height by 13-15% and dry weight by 18%	[44]
	NAIs slightly increased total nitrogen content	[45]
	Lettuce plants exposed to NAIs increased leaf area and fresh weight	[46]
	NAIs improved sprout growth and bacterial control	[47]
	NAIs improved fresh weight, macroelements and microelements in kale	[48]

References:

1. Hawkins, L.H.; Barker, T. Air ions and human performance. *Ergonomics* **1978**, *21*, 273–278, doi:10.1080/00140137808931724.
2. Diamond, M.C.; Connor, J.R.Jr.; Orenberg, E.K.; Bissell, M.; Yost, M.; Krueger, A. Environmental influences on serotonin and cyclic nucleotides in rat cerebral cortex. *Science* **1980**, *210*, 652–654, doi:10.1126/science.6254145.

3. Ju, K.; Kubo, T. Power spectral analysis of autonomic nervous activity in spontaneously hypertensive rats. *Biomed. Sci. Instrum.* **1997**, *33*, 338–343.
4. Ryushi, T.; Kita, I.; Sakurai, T.; Yasumatsu, M.; Isokawa, M.; Aihara, Y.; Hama, K. The effect of exposure to negative air ions on the recovery of physiological responses after moderate endurance exercise. *Int. J. Biometeorol.* **1998**, *41*, 132–136, doi:10.1007/s004840050066.
5. Iwama, H.; Ohmizo, H.; Furuta, S.; Ohmori, S.; Watanabe, K.; Kaneko, T.; Tsutsumi, K. Inspired superoxide anions attenuate blood lactate concentrations in postoperative patients. *Crit. Care Med.* **2002**, *30*, 1246–1249.
6. Iwama, H. Negative air ions created by water shearing improve erythrocyte deformability and aerobic metabolism. *Indoor Air* **2004**, *14*, 293–297, doi:10.1111/j.1600-0668.2004.00254.x.
7. Goel, N.; Terman, M.; Terman, J.S.; Macchi, M.M.; Stewart, J.W. Controlled trial of bright light and negative air ions for chronic depression. *Psychol. Med.* **2005**, *35*, 945–955, doi:10.1017/S0033291705005027.
8. Yamada, R.; Yanoma, S.; Akaike, M.; Tsuburaya, A.; Sugimasa, Y.; Takemiya, S.; Motohashi, H.; Rino, Y.; Takanashi, Y.; Imada, T. Water-generated negative air ions activate NK cell and inhibit carcinogenesis in mice. *Cancer Lett.* **2006**, *239*, 190–197, doi:10.1016/j.canlet.2005.08.002.
9. Sirota, T.V.; Safronova, V.G.; Amelina, A.G.; Mal'tseva, V.N.; Avkhacheeva, N.V.; Sofin, A.D.; Ianin, V.A.; Mubarakshina, E.K.; Romanova, L.K.; Novoselov, VI. Effect of negative air ions on respiratory organs and blood. *Biofizika* **2008**, *53*, 886–893, doi:10.1134/S0006350908050242.
10. Alexander, D.D.; Bailey, W.H.; Perez, V.; Mitchell, M.E.; Su, S. Air ions and respiratory function outcomes: A comprehensive review. *J. Negat. Results Biomed.* **2013**, *12*, 14, doi:10.1186/1477-5751-12-14.
11. Pino, O.; Ragione, F.L. There's something in the air: Empirical evidence for the effects of negative air ions (NAI) on psychophysiological state and performance. *Res. Psychol. Behav Sci.* **2013**, *1*, 48–53.
12. Wiszniewski, A.; Suchanowski, A.; Wielgomas, B. Effects of Air-Ions on human circulatory indicators. *Pol. J. Environ. Stud.* **2014**, *23*, 521–531.
13. Wallner, P.; Kundi, M.; Panny, M.; Tappler, P.; Hutter, H.P. Exposure to air ions in indoor environments: Experimental study with healthy adults. *Int. J. Environ. Res. Public Health.* **2015**, *12*, 14301–14311, doi:10.3390/ijerph121114301.
14. Bowers, B.; Flory, R.; Ametepe, J.; Staley, L.; Patrick, A.; Carrington, H. Controlled trial evaluation of exposure duration to negative air ions for the treatment of seasonal affective disorder. *Psychiatry Res.* **2018**, *259*, 7–14, doi:10.1016/j.psychres.2017.08.040.
15. Bailey, W.H.; Charry, J.M. Acute exposure of rats to air ions: Effects on the regional concentration and utilization of serotonin in brain. *Bioelectromagnetics.* **1987**, *8*, 173–181.
16. Watanabe, I.; Noro, H.; Ohtsuka, Y.; Mano, Y.; Agishi, Y. Physical effects of negative air ions in a wet sauna. *Int. J. Biometeorol.* **1997**, *40*, 107–112, doi:10.1007/s004840050028.
17. McDonald, R.D.; Bachman, C.H.; Lorenz, P.J. Some physiological effects of air ion treatment without ion inhalation. *Int. J Biometeorol.* **1965**, *9*, 141–147, doi:10.1007/bf02188469.
18. Hedge, A.; Collis, M.D. Do negative air ions affect human mood and performance? *Ann. Occup. Hyg.* 1987, 31, 285–290.
19. Reilly, T.; Stevenson, I.C. An investigation of the effects of negative air ions on responses to submaximal exercise at different times of day. *J. Hum. Ergol. (Tokyo).* **1993**, *22*, 1–9.
20. Bailey, W.H.; Williams, A.L.; Leonhard, M.J. Exposure of laboratory animals to small air ions: A systematic review of biological and behavioral studies. *BioMed. Eng. OnLine.* **2018**, *17*, 72, doi:10.1186/s12938-018-0499-z.
21. Phillips, G.; Harris, G.J.; Jones, M.W. Effects of air ions on bacterial aerosols. *Int. J. Biometeorol.* **1964**, *8*, 27–37, doi:10.1007/BF02186925.
22. Kellogg, E.W.; Yost, M.G.; Barthakur, N.; Kreuger, A.P. Superoxide involvement in the bactericidal effects of negative air ions on *Staphylococcus albus*. *Nature* **1979**, *281*, 400–401, doi:10.1038/281400a0.
23. Gabbay, J.; Bergerson, O.; Levi, N.; Brenner, S.; Eli, I. Effect of ionization on microbial air pollution in the dental clinic. *Environ. Res.* **1990**, *52*, 99–106, doi:10.1016/S0013-9351(05)80154-9.
24. Shargawi, J.M.; Theaker, E.D.; Drucker, D.B.; MacFarlane, T.; Duxbury, A.J. Sensitivity of *Candida albicans* to negative air ion streams. *J. Appl. Microbiol.* **1999**, *87*, 889–897, doi:10.1046/j.1365-2672.1999.00944.x.
25. Seo, K.H.; Mitchell, B.W.; Holt, P.S.; Gast, R.K. Bactericidal effects of negative air ions on airborne and surface Salmonella enteritidis from an artificially generated aerosol. *J. Food Prot.* **2001**, *64*, 113–116, doi:10.4315/0362-028X-64.1.113.
26. Noyce, J.O.; Hughes, J.F. Bactericidal effects of negative and positive ions generated in nitrogen on *Escherichia coli*. *J. Electrostatics* **2002**, *54*, 179–187, doi:10.1016/S0304-3886(01)00179-6.
27. Fan, L.; Song, J.; Hildebrand, P.D.; Forney, C.F. Interaction of ozone and negative air ions to control micro-organisms. *J. Appl. Microbiol.* **2002**, *93*, 144–148, doi:10.1046/j.1365-2672.2002.01683.x.
28. Noyce, J.O.; Hughes, J.F. Bactericidal effects of negative and positive ions generated in nitrogen on starved *Pseudomonas veronii*. *J. Electrostatics* **2003**, *57*, 49–58, doi:10.1016/S0304-3886(02)00110-9.
29. Kerr, K.G.; Beggs, C.B.; Dean, S.G.; Thornton, J.; Donnelly, J.K.; Todd, N.J.; Sleigh, P.A.; Qureshi, A.; Taylor, C.C. Air ionisation and colonization / infection with methicillin-resistant *Staphylococcus aureus* and *Acinetobacter* species in an intensive care unit. *Intensive Care Med.* **2006**, *32*, 315–317, doi:10.1007/s00134-005-0002-8.
30. Fan, X.; Fett, W.F.; Mitchell, B.W. Effect of negative air ions on *Escherichia coli* ATCC 25922 inoculated onto mung bean seed and apple fruit. *J. Food Prot.* **2007**, *70*, 204–208, doi:10.4315/0362-028X-70.1.204.

31. Fletcher, L.A.; Gaunt, L.F.; Beggs, C.B.; Shepherd, S.J.; Sleigh, P.A.; Noakes, C.J.; Kerr, K.G. Bactericidal action of positive and negative ions in air. *BMC Microbiol.* **2007**, *7*, 32, doi:10.1186/1471-2180-7-32.
32. Tyagi, A.K.; Nirala, B.K.; Malik, A.; Singh, K. The effect of negative air ion exposure on Escherichia coli and Pseudomonas fluorescens. *J. Environ. Sci. Health A Tox. Hazard Subst. Environ. Eng.* **2008**, *43*, 694–699, doi:10.1080/10934520801959831.
33. Escombe, A.R.; Moore, D.A.; Gilman, R.H.; Navincopa, M.; Ticona, E.; Mitchell, B.; Noakes, C.; Martínez, C.; Sheen, P.; Ramirez, R.; et alUpper-room ultraviolet light and negative air ionization to prevent tuberculosis transmission. *PLoS Med.* **2009**, *6*, e43, doi:10.1371/journal.pmed.1000043.
34. Kampmann, Y.; Klingshirn, A.; Kloft, K.; Kreyenschmidt, J. The application of ionizers in domestic refrigerators for reduction in airborne and surface bacteria. *J. Appl. Microbiol.* **2009**, *107*, 1789–1798, doi:10.1111/j.1365-2672.2009.04359.x.
35. Tyagi, A.K.; Malik, A. Antimicrobial action of essential oil vapours and negative air ions against Pseudomonas fluorescens. *Int. J. Food Microbiol.* **2010**, *143*, 205–210, doi:10.1016/j.ijfoodmicro.2010.08.023.
36. Dobrynin, D.; Friedman, G.; Fridman, A.; Starikovskiy, A. Inactivation of bacteria using DC corona discharge: Role of ions and humidity. New J. Phys. **2011**, pii: 103033, doi:10.1088/1367-2630/13/10/103033.
37. Timoshkin, I.V.; Maclean, M.; Wilson, M.P.; Given, M.J.; MacGregor, S.J.; Wang, T.; Anderson, J.G. Bactericidal effect of corona discharges in atmospheric air. *IEEE T. Plasma Sci.* **2012**, *40*, 2322–2333, doi:10.1109/TPS.2012.2193621.
38. Zhou, P.; Yang, Y.; Huang, G.; Lai, A.C.K. Numerical and experimental study on airborne disinfection by negative ions in air duct flow. *Build. Environ.* **2018**, *127*, 204–210, doi:10.1016/j.buildenv.2017.11.006.
39. Pratt, R.; Barnard, R.W. Some effects of ionized air on *Penicillium notatum*. *J. Am. Pharm. Assoc. Sci.* **1960**, *49*, 643–646, doi:10.1002/jps.3030491004.
40. Mitchell, B.W.; King, D.J. Effect of negative air ionization on airborne transmission of newcastle disease virus. *Avian. Dis.* **1994**, *38*, 725–732, doi:10.2307/1592107.
41. Krueger, A.P.; Kotaka, S.; Andriese, P.C. Studies on the Effects of Gaseous Ions on Plant Growth I. The influence of positive and negative air ions on the growth of *Avena sativa*. *J. Gen Physiol.* **1962**, *45*, 879–895, doi:10.1085/jgp.45.5.879.
42. Krueger, A.P.; Kotaka, S.; Andriese, P.C. Some observations on the physiological effects of gaseous ions. *Int. J. Biometeorol.* **1962**, *6*, 33–48, doi:10.1007/BF02187011.
43. Kotaka, S.; Krueger, A.P.; Andriese, P.C.; Nishizawa, K.; Ohuchi, T.; Takenobu, M.; Kozure, Y. Air ion effects on the oxygen consumption of barley seedlings. *Nature* **1965**, *208*, 1112–1113, doi:10.1038/2081112a0.
44. Elkiey, T.M.; Bhartendu, S.; Barthakur, N. Air ion effect on respiration and photosynthesis of barley and *Antirrhinum majus*. *Int. J. Biometeorol.* **1985**, *29*, 285–292, doi:10.1007/BF02189659.
45. Barthakur, N.N.; Arnold, N.P. Growth and certain chemical constituents of tobacco plants exposed to air ions. *Int. J. Biometeorol.* **1988**, *32*, 78–80, doi:10.1007/BF01044897.
46. Song, M.J.; Kang, T.H.; Han, C.S.; Oh, M.M. Air anions enhance lettuce growth in plant factories. *Hortic. Environ. Biotechnol.* ***2014***, *55*, 293–298, doi:10.1007/s13580-014-1016-3.
47. Song, M.J.; Jeon, Y.M.; Oh, M.M. Growth of and bacterial counts on several edible sprouts exposed to spray ionization. *Protected Hort. Plant Fac.* **2015**, *24*, 45–50, doi:10.12791/KSBEC.2015.24.1.045.
48. Lee, S.R.; Kang, T.H.; Han, C.S.; Oh, M.M. Air anions improve growth and mineral content of kale in plant factories. *Hortic. Environ. Biotechnol.* **2015**, *56*, 462–471, doi:10.1007/s13580-015-0035-z.
